# Insights into *Arcanobacterium haemolyticum*: A Narrative Review of an Emerging Pathogen Revisited

**DOI:** 10.3390/pathogens15030335

**Published:** 2026-03-21

**Authors:** Alessandra Consonni, Elena Briozzo, Chiara Giubbi, Silvia Tonolo, Francesco Luzzaro, Carola Mauri

**Affiliations:** Clinical Microbiology and Virology Unit, “A. Manzoni” Hospital, 23900 Lecco, Italy

**Keywords:** pharyngitis, narrative review, Lemierre’s syndrome, soft tissue infections, bacteremia

## Abstract

*Arcanobacterium haemolyticum* is a facultative anaerobic, Gram-positive bacillus that has garnered attention due to its role in human infections, particularly among adolescents and young adults. Traditionally associated with pharyngitis, this organism is increasingly recognized for its involvement in systemic infections, including bacteremia, central nervous system abscesses, and Lemierre’s syndrome. The pathogenicity of *A. haemolyticum* is attributed to its production of hemolysins and neuraminidase, facilitating tissue invasion and immune evasion. Clinically, infections often present with sore throat, fever, and a characteristic scarlatiniform rash, which can lead to their misdiagnosis as streptococcal pharyngitis. Severe manifestations, though rare, have been documented, particularly in immunocompromised individuals. Diagnosis is challenging due to the organism’s slow growth and potential misidentification as diphtheroids in cultures. Accurate identification necessitates specific culture conditions and biochemical testing. Treatment typically involves beta-lactam antibiotics; however, the emergence of resistance patterns necessitate susceptibility testing to guide therapy. This review aims to consolidate current knowledge on *A. haemolyticum*, emphasizing its clinical presentations, diagnostic challenges, and management strategies, thereby enhancing recognition and treatment of infections caused by this emerging pathogen.

## 1. Introduction

*Arcanobacterium haemolyticum,* formerly known as *Corynebacterium haemolyticum,* is a facultative anaerobic, Gram-positive bacterium recognized for its clinical significance as a causative agent of pharyngitis, wound infections, and, less frequently, invasive diseases [1]. This bacterium, belonging to the genus *Arcanobacterium*, exhibits unique phenotypic and genotypic characteristics that distinguish it from other related species, contributing to its specific pathogenic mechanisms and clinical presentations.

*A. haemolyticum* is most commonly implicated in infections of the head and neck, particularly acute pharyngitis and sinusitis in children and adolescents. It is also a recognized cause of skin and soft-tissue infections, especially in immunocompromised individuals. Although less frequently encountered, the organism has been documented in a range of invasive infections, including bacteremia, endocarditis, osteomyelitis, severe sepsis, brain abscesses, and pneumonia [2,3,4]. Distinguishing the etiology of acute pharyngitis can be challenging in the absence of microbiological confirmation, as clinical features alone are often insufficient to differentiate bacterial from viral causes. *Streptococcus pyogenes* remains the most common bacterial pathogen in this context; however, *A. haemolyticum* is responsible for up to 2.5% of cases [5]. The clinical presentation of these two bacterial infections is often remarkably similar, underscoring the importance of considering *A. haemolyticum* in the differential diagnosis to ensure accurate identification and timely intervention. The manifestations of *A. haemolyticum* pharyngitis typically include sore throat, fever, and a pruritic, “sandpaper-like” scarlatiniform rash. This rash may closely resemble the cutaneous findings of scarlet fever caused by *S. pyogenes*, leading to potential misclassification. Furthermore, the rash may be mistaken for a viral exanthem, adding to the diagnostic complexity [6,7]. Delays in diagnosis are further compounded by laboratory factors: β-hemolysis produced by *A. haemolyticum* generally becomes evident only after 48–72 h of incubation, in contrast to the more rapid—often within 24 h—hemolytic pattern of *S. pyogenes*. In addition, *A. haemolyticum* is inherently more difficult to isolate in culture, creating further obstacles to prompt microbiological confirmation [2]. Early recognition is crucial, as timely initiation of antimicrobial therapy significantly improves outcomes. Effective treatment typically involves penicillins, macrolides, or tetracyclines, administered alongside appropriate supportive care. When diagnosed and managed promptly, patients generally recover fully without long-term sequelae.

Understanding the multifaceted nature of *A. haemolyticum* requires a comprehensive review of its microbiological properties, pathogenic mechanisms, clinical manifestations, diagnostic approaches, and therapeutic strategies, providing a foundation for improved clinical management and future research directions. The increasing prevalence of antibiotic resistance among bacterial pathogens underscores the importance of understanding the intricacies of bacterial pathogenesis and developing effective strategies for combating infections. Furthermore, insights into the virulence factors and host interactions of *A. haemolyticum* are crucial for designing targeted therapies and preventive measures. In this review, we aim to synthesize current knowledge regarding *A. haemolyticum*, offering a holistic perspective on its role in human health and disease.

## 2. Materials and Methods

### 2.1. Search Strategy and Inclusion and Exclusion Criteria

This narrative review aims to summarize all the data on *A. haemolyticum* infections in humans published in the literature in the last 20 years. Information regarding patients’ demographics, clinical characteristics, site of infection, clinical presentation, and treatment provided were described. There was a particular focus on data regarding microbiological characteristics and methodologies used for identification and antimicrobial susceptibility testing (AST).

By using the PubMed/Medline and Embase database, searches for relevant articles were performed with the following item: “(*Arcanobacterium haemolyticum* infection)”. Studies providing original data, such as case reports and letters to the editor providing information on *A. haemolyticum* infections in humans, were included in the review. Searches were limited to articles published in English from 1 January 2005 up to 31 December 2025. Studies regarding infections and/or colonization in animals were excluded from the analysis. Moreover, the reference lists of reports identified by this search strategy were also hand-searched to select further relevant articles.

### 2.2. Data Extraction

After the initial screening, the following data were extracted from each included study: publication year, age and gender of patients, type of infection, underlying disease and antibiotic therapy. Other relevant microbiological data were collected: type of sample, method of identification, antimicrobial susceptibility testing profile and co-isolated bacteria.

## 3. Results

### 3.1. Literature Search and Clinical Information

The literature search yielded a total of 187 records (PubMed, *n* = 81; Embase, *n* = 106). After removal of 54 duplicates, 133 articles were screened based on title and abstract, leading to the exclusion of 61 records. Of the remaining 72 full-text articles assessed for eligibility, 21 were excluded for the following reasons: article not published in English (*n* = 10), review articles or responses to letters (*n* = 6), and reports describing infections in animals (*n* = 5). Overall, 51 studies met the inclusion criteria and were included in the qualitative synthesis. These publications comprised case reports, case series, and letters to the editor, describing a total of 73 individual cases of *A. haemolyticum* infection in humans.

The main demographic and clinical features of *A. haemolyticum* infections reported in the literature are summarized in Table 1. Among the reported cases, 50 patients were male and 23 were female, resulting in male predominance. Patient age ranged from 2 to 91 years, with a mean age of 44.5 years and an IQR of 21–64 (M: 20.3–61.5; F: 31–66.5). A wide spectrum of clinical manifestations was observed. Bloodstream infections and skin and soft tissue infections were the most frequently reported presentation (*n* = 19), followed by brain abscess and Lemierre’s syndrome (*n* = 6). Bone infections (*n* = 4), intra-abdominal infections (*n* = 4), and respiratory tract infections (*n* = 4) were less common. Pyothorax (*n* = 3), endocarditis (*n* = 2), and urinary tract infection (*n* = 1) were rarely reported. When multiple infectious foci were present, classification was based on the most severe infection requiring treatment. Regarding microbiological context, infections were monomicrobial in 38 samples, whereas 32 samples were polymicrobial. In polymicrobial infections, *A. haemolyticum* was most frequently co-isolated with β-hemolytic streptococci (*n* = 16), anaerobic bacteria (*n* = 12), and *Staphylococcus aureus* (*n* = 10), supporting a potential synergistic role in mixed infections.

### 3.2. Phylogenetic of A. haemolyticum

The genus *Arcanobacterium* was first described by Collins et al. [8] to accommodate the species formerly known as *Corynebacterium haemolyticum*, initially classified by MacLean et al. (1946) [9]. The delineation of this new genus was based on both morphological features and chemotaxonomic criteria, including the composition of the cell wall peptidoglycan, the presence of specific long-chain fatty acids, and the profile of respiratory quinones. Over time, the genus expanded to include nine validly published species: *A. haemolyticum*, *A. pyogenes*, *A. bernardiae*, *A. bialowiezense*, *A. bonasi*, *A. abortisuis*, *A. hippocoleae*, *A. phocae*, and *A. pluranimalium*. Some of these, including *A. pyogenes* and *A. bernardiae*, were transferred from other genera such as *Actinomyces* and *Corynebacterium*, reflecting earlier misclassifications. Notably, *A. pyogenes* originated as ‘*Corynebacterium pyogenes*’, then ‘*Actinomyces pyogenes*’, and was eventually assigned to *Arcanobacterium* by Ramos et al. (1997) [10]. These transfers were based largely on phenotypic similarities and early chemotaxonomic observations. However, emerging evidence began to challenge the coherence of this taxonomic framework. Lehnen et al. (2006) [11] proposed that only *A. haemolyticum*, *A. phocae*, *A. pluranimalium*, and *A. hippocoleae* should be retained within *Arcanobacterium*, while *A. pyogenes*, *A. bernardiae*, *A. bialowiezense*, and *A. bonsai* should be reassigned to a new genus. To test the validity of the proposed subdivision, comprehensive phylogenetic analyses based on 16S rRNA gene sequences were undertaken. These molecular data were systematically compared with extended chemotaxonomic profiles—including fatty acid compositions, polar lipid profiles, cell-wall sugar types, acyl groups of muramic acid residues, peptidoglycan structure, and DNA G + C content—for all nine species within the original *Arcanobacterium* designation. The integration of these data confirmed a clear dichotomy within the genus, substantiating the earlier hypothesis of polyphyly. Species such as *A. haemolyticum*, *A. hippocoleae*, *A. phocae*, and *A. pluranimalium* formed a distinct clade consistent with the original definition of *Arcanobacterium*, while *A. pyogenes*, *A. bernardiae*, *A. bialowiezense*, and *A. bonasi* were phylogenetically divergent and chemotaxonomically distinct. This comprehensive evidence ultimately justified the establishment of a novel genus, *Trueperella*, to house the latter group. The reclassification of multiple *Arcanobacterium* species into the new genus *Trueperella* represents a necessary refinement in the taxonomy of the family *Actinomycetaceae*, grounded in molecular phylogenetics and robust chemotaxonomic data. The genus *Arcanobacterium*, now limited to its core species, is re-affirmed as a coherent phylogenetic unit, while *Trueperella* accommodates a distinct evolutionary lineage with demonstrably different biochemical and genomic features.

### 3.3. Etiology and Pathogenesis of A. haemolyticum

*A. haemolyticum* is a catalase-negative, aerobic, β-hemolytic, non-motile, branching, Gram-positive bacillus that forms part of the normal flora of the skin and nasopharynx. It is notable for its ability to inhibit hemolysis by *S. aureus* in the Christie–Atkins–Munch–Peterson (CAMP) test, while enhancing hemolysis by *Streptococcus agalactiae* in the reverse CAMP test [1,2]. *A. haemolyticum* is primarily transmitted via direct person-to-person contact through respiratory droplets, with detection in the oropharynx of household contacts supporting probable intrafamilial transmission [7]. Two phenotypic biotypes have been described: smooth and rough. Smooth biotypes are more frequently associated with soft-tissue infections and form uniform, β-hemolytic colonies on solid media. They are β-glucuronidase negative and capable of fermenting sucrose and trehalose. In contrast, rough biotypes—commonly linked to pharyngitis—produce uneven, non-hemolytic colonies, are β-glucuronidase positive, and do not ferment sucrose or trehalose [12]. On blood agar, *A. haemolyticum* produces β-hemolytic colonies composed of Gram-positive bacilli, with optimal growth at 37 °C in an atmosphere containing 5–10% CO_2_. Hemolysis is most prominent on human or horse blood agar; however, growth on sheep blood agar may be slower, with hemolysis becoming evident only after up to 72 h of incubation [13]. This delay may result in false-negative cultures unless extended observation is specifically requested. Definitive identification is achieved through demonstration of catalase negativity and a positive CAMP test. Misidentification can occur in early culture stages, as colony morphology may resemble normal flora or other organisms, including *Corynebacterium* spp., *Streptococcus* spp., and *A. pyogenes*. Differentiation from *Corynebacterium* spp. is based on β-hemolysis in combination with catalase negativity; from *A. pyogenes*, based on the inability to ferment xylose and a positive reverse CAMP reaction; and from *Streptococcus* spp., based on distinct microscopic morphology [2].

The pathogenesis of *A. haemolyticum* is multifactorial, involving both bacterial virulence factors and host predispositions. Experimental evidence highlights the role of hemolysins and neuraminidase in promoting tissue damage, adherence, and immune evasion, which underlies the scarlatiniform rash and invasive potential observed clinically. Case-based evidence further illustrates that *A. haemolyticum* infections often arise in otherwise healthy young individuals with pharyngitis and peritonsillar abscesses, but severe manifestations—including intracerebral abscesses, Lemierre’s syndrome, necrotizing fasciitis, endocarditis, and bacteremia—occur more frequently in patients with comorbidities such as diabetes mellitus, cardiac valve disease, or immunosuppression (Table 2) [14,15,16,17,18]. Co-infections with organisms such as *S. agalactiae*, *S. dysgalactiae*, *S. aureus*, *Fusobacterium necrophorum*, and other anaerobic bacteria are commonly reported and may act synergistically, worsening the clinical course (Table 1 and Table 3). The ability of *A. haemolyticum* to cause both localized suppurative infections (e.g., soft-tissue abscesses, orbital cellulitis) and disseminated disease (e.g., sepsis, meningitis) reflects its capacity for tissue invasion and survival in blood and deep compartments (Table 2). Limited information is available regarding the virulence factors of *A. haemolyticum*, and as a result, the mechanisms underlying pharyngeal infection and subsequent spread into deeper tissues remain poorly defined. Early investigations into virulence involved intradermal inoculation of the bacterium into humans, guinea pigs, and rabbits, which produced raised abscesses characterized by necrosis and marked neutrophil infiltration within 24–48 h post-infection. Intravenous administration of *A. haemolyticum* into rabbits induced hemorrhagic pneumonia [9], indicating that the organism is capable of causing invasive disease once disseminated through the bloodstream. Later, a phospholipase D (PLD) was identified and implicated in the dermonecrotic lesions observed [19]. Although the precise contribution of *A. haemolyticum* PLD to pathogenesis remains unresolved, its expression during infection has been demonstrated through the detection of serum antibodies in patients with pharyngitis [20,21]. PLDs are widely distributed enzymes that hydrolyze phospholipids such as phosphatidylcholine and sphingomyelin, both of which are abundant in mammalian plasma membranes. Sphingomyelin, together with cholesterol and GPI-anchored proteins, preferentially localizes to lipid rafts—highly ordered membrane microdomains that compartmentalize cellular processes on the outer plasma membrane leaflet [22]. These lipid rafts have also been implicated in microbial invasion of host cells. One molecular mechanism suggested that PLD may be essential for optimal adhesion of the bacterium to host cells through the remodeling of lipid rafts. Moreover, intracellular expression of PLD exerts a direct cytotoxic effect, inducing host cell death via necrosis. In detail, host-derived PLD cleaves sphingomyelin, resulting in the release of ceramide. The accumulation of ceramide within lipid rafts alters their biophysical characteristics, promoting the formation of large, ceramide-enriched membrane platforms [23]. These specialized domains enable the reorganization and clustering of protein receptors and receptor-associated signaling molecules, thereby enhancing the efficiency of signal transduction required for normal physiological functions. *A. haemolyticum* PLD shares the closest homology with the PLD of *Corynebacterium pseudotuberculosis* [24]. In *C. pseudotuberculosis*, PLD is essential for virulence, as mutants lacking the *pld* gene are unable to disseminate from the initial site of inoculation or persist within lymph nodes [25]. The PLD produced by *C. pseudotuberculosis* hydrolyzes sphingomyelin in host cell membranes and lysophosphatidylcholine in plasma, resulting in endothelial membrane disruption and cytolysis, which collectively enhance vascular permeability [25]. Additionally, *C. pseudotuberculosis* PLD has been shown to activate complement [26], induce neutrophil chemotaxis [27], and cause direct dermonecrosis upon intradermal injection.

**Table 2:** Clinical features of *Arcanobacterium haemolyticum* infections reported in the literature.

Author Year Reference	Type of Infection	Underlying Illness and Risk Factors and Medical History	Co-Isolated Bacteria	Treatment	Outcome
Chinello (2025) [28]	Orbital cellulitis and intracerebral abscess	No underlying disease or history of interest.	No	Carbapenem, cephalosporin, daptomycin, lincosamide, oxazolidinone, penicillin with β-lactamase inhibitors	Complete resolution of the infection
Faiz (2025) [29]	Appendicitis	No underlying disease History of cocaine use.	No	Cephalosporin, metronidazole and tetracycline	Complete resolution of the infection
Lovering (2025) [30]	Chronic osteomyelitis and bacteremia	Paraplegia with neurogenic bladder, legs amputation and chronic stage IV ischial decubitus ulcer.	No	Glycopeptide and penicillin with β-lactamase inhibitors	Complete resolution of the infection
Saijo (2025) [31]	Necrotizing fasciitis	Poorly controlled diabetes mellitus.	*Streptococcus agalactiae*	Lincosamide and penicillin with β-lactamase inhibitors	Resolution of the infection after amputation and more than 30 days of therapy
Bozso (2024) [32]	Bacteraemia with possible meningitis	Myxomatous mitral valve and moderate mitral valve regurgitation and prolapse.	No	Cephalosporin and fluoroquinolone	Complete resolution of the infection
Chang (2024) [33]	Intracerebral abscess	No underlying disease or history of interest.	*Fusobacterium* (blood culture) *Streptococcus anginosus* (intraoperative samples)	Cephalosporin, glycopeptide, macrolide, metronidazole, penicillin	Complete resolution of the infection
Chin (2024) [34]	Orbital abscess and cerebral ischemia	No underlying disease or history of interest.	No	Multiple antibiotic therapy (not specified)	Resolution of the infection (persistence of hemiparesis)
Grusiecki (2024) [35]	Peritonsillar abscess	No underlying disease or history of interest.	No	Azole, cephalosporin, lincosamide, metronidazole, penicillin, penicillin with β-lactamase inhibitors	Resolution of the infection after multiple antibiotic therapy
Gu (2024) [36]	Soft-tissue infection	No underlying disease or history of interest.	*Streptococcus dysgalactiae*	Azole, cephalosporin and mupirocin	At the 2-week follow-up, the patient had near-complete resolution of his cutaneous changes.
Lampejo (2024) [37]	Lemierre’s syndrome	NR	No	Cephalosporin, metronidazole and penicillin with β-lactamase inhibitors	NR
Alrwashdeh (2023) [38]	Bacteremia secondary to peritonsillar abscess	History of epilepsy (in treatment with levetiracetam). Remote history of a gunshot wound to his abdomen. Use of marijuana and alcohol consumption.	NR	Cephalosporin, glycopeptide and penicillin with β-lactamase inhibitors	Patient discharged after over a month. Healthy at the follow-up visit after 2 months.
Herai (2023) [39]	Pyothorax	Cerebral infarction and Alzheimer’s disease; diabetes mellitus; foot ulcer.	*Staphylococcus aureus*	Cephalosporin, lincosamide and penicillin with β-lactamase inhibitors	Complete resolution of the infection
Sahhar (2023) [40]	Intracerebral abscess (subdural empyema) caused by invasive sinusitis	No underlying disease or history of interest.	*F. necrophorum*	Cephalosporin, glycopeptide and metronidazole	Death
Hicks (2022) [41]	Necrotizing fasciitis	Homeless, drug user, history of HCV, malnourishment. No diabetes or HIV.	No	Lincosamide, penicillin, penicillin with β-lactamase inhibitors	Complete resolution of the infection
Thomas (2022) [42]	Necrotic wound on left foot	Diabetes.	No	Fluoroquinolone	NR
	Wound infection	Diabetes and hypertension.	No	Lincosamide and fluoroquinolone	NR
	Wound gangrene infection	Diabetes and Non-ST elevation; myocardial infarction.	No	Glycopeptide and penicillin with β-lactamase inhibitors	NR
	Necrotic wound infection	Diabetes, hypertension, and hypothyroidism.	Β-hemolytic streptococci	Lincosamide and fluoroquinolone	NR
	Wound infection following trauma	No known comorbidities.	*S. aureus*	Fluoroquinolone and metronidazole	NR
	Ulcer infection following injury	Diabetes.	*Proteus penneri*	Lincosamide and fluoroquinolone	NR
Adams (2020) [43]	Sinusitis (after pharyngitis) complicated by preseptal cellulitis and cerebral abscess	No underlying disease or history of interest.	No	Cephalosporin, glycopeptide and metronidazole	Complete resolution of the infection
Saeb (2021) [44]	Chronic wound infection and osteomyelitis	Type 2 diabetes for 32 years, severe bilateral neuropathy involving both lower extremities and bilateral background retinopathy.	*S. aureus*	Fluoroquinolone and penicillin	Complete resolution of the infection
Lobo (2020) [45]	Liver abscess	NR	No	Carbapenem and metronidazole	Complete resolution of the infection
Verona (2020) [46]	Bacteraemia	No underlying disease or history of interest.	No	Cephalosporin, glycopeptide and penicillin with β-lactamase inhibitors	Patient discharged after over 19 days. Healthy at the follow-up visit.
Takamura (2019) [3]	Left heel ulcer, osteomyelitis and bacteraemia	Hypertension and left leg deep vein thrombosis.	*S. dysgalactiae*	Penicillin with β-lactamase inhibitors	Complete resolution of the infection
Poplin (2018) [47]	Brain abscess and subdural empyema	No underlying disease. EBV infection.	No	Cephalosporin, glycopeptide, metronidazole and tetracycline	Complete resolution of the infection
Seki (2019) [48]	Intrathoracic abscess	Uncontrolled diabetes.	No	Penicillin with β-lactamase inhibitors	NR
Cortés-Penfield (2017) [49]	Intracranial abscess and bacteraemia	No underlying disease or history of interest.	Blood culture monomicrobial. Intraoperative samples: no growth. PCR 16s rRNA positive also for *Bacteroides* spp., *Anaerococcus tetradius*, *Dialister micraerophilus*, *Erysipelotrichaceae* spp., and *Propionibacterium acnes*	Cephalosporin, glycopeptide, metronidazole and penicillin with β-lactamase inhibitors	Complete resolution of the infection
Smith (2016) [50]	Ulcer and bacteraemia	Uncontrolled diabetes, hypertension, dyslipidemia and osteomyelitis (amputation 1 year previously).	Blood culture monomicrobial. *Enterococcus avium* (wound swab)	Daptomycin, fluoroquinolone and penicillin with β-lactamase inhibitors	NR
Miyamoto (2015) [4]	Calf cellulitis	Non-Hodgkin’s malignant lymphoma in the neck.	*S. dysgalactiae*, *Corynebacterium striatum*	NR	NR
	Foot, former cellulitis	Brainstem infarction post-treatment for laryngeal cancer.	*S. dysgalactiae*, *C. striatum*, *S. aureus*	NR	NR
	Leg ulcer	Uncontrolled diabetes with gangrene in the foot.	*S. dysgalactiae*, *C. striatum*, *Peptoniphilus asaccharolyticus*	NR	NR
	Femoral ulcer	T2 squamous cell carcinoma in the femur.	Meticillin-resistant *S. aureus*	NR	NR
	Pressure sore in buttocks	Quadriplegia	*S. dysgalactiae*, *Streptococcus* *agalactiae*, *Streptococcus anginosus* group, *Peptoniphilus anaerobius*	NR	NR
	Femoral ulcer	Primary hypertension post-surgery for an Achilles tendon rupture.	*S. dysgalactiae*, *S.* *agalactiae*, *S. aureus*, *S. anginosus* group, *Anaerococcus prevotii*	NR	NR
	Calf cellulitis	Articular rheumatism osteoarthrosis.	*C. striatum*, *Prevotella disiens*, *Finegoldia magna*	NR	NR
Stone (2015) [51]	Orbital necrotizing fasciitis and osteomyelitis	No underlying disease or history of interest.	No	Lincosamide, glycopeptide and penicillin with β-lactamase inhibitors	Complete resolution of the infection
Brown (2013) [52]	Soft-tissue infection and sepsis	Hypertension, history of left-sided Charcot foot.	Blood culture monomicrobial. Group B β-hemolytic streptococci in intraoperative sample	Penicillin with β-lactamase inhibitors	Complete resolution of the infection
Ji (2013) [53]	Lemierre’s syndrome	Smoker. No underlying disease or history of interest.	No	Carbapenem, glycopeptide, macrolide, penicillin and penicillin with β-lactamase inhibitors	Complete resolution of the infection
Ramey (2013) [14]	Orbital cellulitis and bacteraemia	No underlying disease or history of interest. Diagnosis of EBV infectious mononucleosis.	*Fusobacterium* species in blood culture. Coagulase-negative staphylococci in abscess culture	Cephalosporin, glycopeptide, macrolide, metronidazole, penicillin and penicillin with β-lactamase inhibitors	Complete resolution of the infection
Alatoom (2012) [54]	Endocarditis	Hypertension, non-insulin-dependent diabetes mellitus, depression, atherosclerotic cardiovascular disease, congenital bicuspid aortic valve, smoker.	No	Macrolide	Death
Hedman (2012) [15]	Septicemia	No underlying disease. Two years before: surgery of the arch of the foot with osteosynthesis to treat pes planus.	*S. aureus* in wound swab. Blood culture monomicrobial	Cephalosporin and macrolide	Complete resolution of the infection
	Septicemia	No underlying disease or history of interest.	*F. necrophorum*	NR	NR
	Septicemia	Immunocompromised with multiple diseases, including diabetes mellitus, poor dental status and foot wounds.	No	NR	NR
Lee (2012) [55]	Lemierre’s syndrome	No underlying disease or history of interest.	No	Aminoglycoside, cephalosporin, glycopeptide and metronidazole	Complete resolution of the infection
Sayyahfar (2012) [56]	Thyroid abscess	No underlying disease or history of interest.	No	Cephalosporin and lincosamide	Complete resolution of the infection
Ribakovs (2011) [57]	Spinal infections and severe sepsis	Two weeks before: anal resection of a malignant rectal polyp.	No	Penicillin	NR
Saxena (2011) [58]	Chorioamnionitis	No underlying disease. Primigravida at 32 weeks of gestation.	No	Aminoglycoside and metronidazole	Complete resolution of the infection (patient was induced and a healthy baby was delivered)
Wong (2011) [59]	Endocarditis complicated with cerebral emboli	Congenital heart disease. Surgical repair of pulmonary atresia, quadricuspid aortic valve and a ventricula septal defect.	No	Aminoglycoside, cephalosporin, glycopeptide, metronidazole	Complete resolution of the infection
Lundblom (2010) [60]	Lemierre’s syndrome	No underlying disease or history of interest.	*F. necrophorum*	Aminoglycoside cephalosporin, macrolide and penicillin with β-lactamase inhibitors	Complete resolution of the infection
Fernández-Suárez (2009) [16]	Lemierre’s syndrome	No underlying disease or history of interest.	No	Carbapenem, lincosamide, macrolide, Metronidazole, and penicillin with β-lactamase inhibitors	Complete resolution of the infection
Aracil (2008) [61]	Osteomyelitis	No underlying disease. Smoker and onychophagia.	No	Cephalosporin	Resolution of the infection
Lee (2008) [17]	Dog-bite induced necrotizing fasciitis	Uncontrolled diabetes.	*F. magna* and *S. agalactiae*	Carbapenem, glycopeptide, lincosamide, penicillin	Complete resolution of the infection
Malini (2008) [62]	Soft-tissue infection	No underlying disease or history of interest.	Group G β-hemolytic streptococci	Cephalosporin, macrolide	Complete resolution of the infection
	Soft-tissue infection and osteomyelitis	Diabetes under treatment for five years.	*Proteus vulgaris*	Macrolide and penicillin	NR
	Soft-tissue infection	No underlying disease or history of interest.	Group C β-hemolytic streptococci	Fluoroquinolone and penicillin	Complete resolution of the infection
Therriault (2008) [1]	Pneumonia and severe sepsis	Mild asthma.	No	Carbapenem, fluoroquinolone, glycopeptide, macrolide, penicillin, penicillin with β-lactamase inhibitors and trimethoprim–sulfamethoxazole	Complete resolution of the infection
Volante (2008) [18]	Sinusitis	NR	No	Cephalosporin, penicillin	Complete resolution of the infection
	Pharyngitis	NR	No	Cephalosporin and penicillin with β-lactamase inhibitors	Complete resolution of the infection
Schroeder (2007) [63]	Lemierre’s syndrome	No underlying disease. Use of marijuana and tobacco, alcohol consumption.	No	Cephalosporin, metronidazole and penicillin with β-lactamase inhibitors	Complete resolution of the infection
van Loo (2007) [64]	Pelvic abscess	No underlying disease. History of a membranous glomerulopathy in 2004, with complete recovery.	No	Macrolide	Complete resolution of the infection
Ciraj (2006) [65]	Urinary tract infection	NR	No	Penicillin	Complete resolution of the infection
Farmer (2007) [66]	Soft-tissue infection and bacteremia	Bed-bound patient with ulcerated pressure areas and nasogastric feeding regime. History of anemia and recurrent urinary tract infection.	*Bacteroides fragilis* (blood culture) *Pseudomonas aeruginosa* and methicillin-resistant *Staphylococcus aureus* (ulcer culture).	Penicillin with β-lactamase inhibitors	NR
Tan (2006) [67]	Soft-tissue infection and bacteremia	Ischemic heart disease, hypertension and poorly controlled diabetes mellitus.	Blood culture monomicrobial. *P. aeruginosa*, group G β-hemolytic streptococci and mixed anaerobes (abscess culture).	Fluoroquinolone and penicillin	Clinical improvement after antibiotic therapy
	Soft-tissue infection and bacteremia	Insulin-dependent diabetes mellitus, hypertension, hyperlipidemia and previous metatarsal amputations following infection.	Blood culture monomicrobial. *S. aureus* and group G β-hemolytic streptococci (abscess culture).	Cephalosporin and metronidazole	NR
	Soft-tissue infection and bacteraemia	Insulin-dependent diabetes mellitus, complicated by hyperlipidemia, peripheral neuropathy, diabetic nephropathy and retinopathy.	No	Penicillin and penicillin with β-lactamase inhibitors	Complete resolution of the infection
	Soft-tissue infection and bacteraemia	Bed-bound patient and nasogastric feeding regime. History of multiple infarct cerebral disease, hypertension and epilepsy.	Blood culture monomicrobial. *S. aureus* and group G β-hemolytic streptococci (ulcer culture).	Penicillin and penicillin with β-lactamase inhibitors	Complete resolution of the infection
	Spontaneous bacterial peritonitis	No underlying disease or history of interest.	No	Macrolide	Complete resolution of the infection
Vargas (2006) [68]	Brain abscess (after dental extraction procedure)	No underlying disease or history of interest. Repeated periodontal manipulations.	No	Cephalosporin, metronidazole and penicillin	Complete resolution of the infection
Goyal (2005) [69]	Septic arthritis	No underlying disease Osteoarthritis for two last years	No	Aminoglycoside and penicillin with β-lactamase inhibitors	Complete resolution of the infection
Katkar (2005) [70]	Respiratory tract infection	No underlying disease or history of interest.	*Mycobacterium tuberculosis*	Aminoglycoside and penicillin	Complete resolution of the infection
Parija (2005) [71]	Pyothorax	No underlying disease or history of interest.	No	Cephalosporin and metronidazole	Complete resolution of the infection
Varma (2005) [72]	Chronic canaliculitis	No underlying disease. Four-month history of painful upper lid swelling and discharge from the left eye.	No	Penicillin	Complete resolution of the infection

NR: not reported.

**Table 3:** Laboratory identification and clinical management of *Arcanobacterium haemolyticum* infections reported in the literature.

Author Year Reference	Type of Sample	Method of Identification	Method of Susceptibility Testing	Interpretation Criteria	Antibiotics Resistance Phenotype (MIC mg/L)
Chinello (2025) [28]	Material drained from orbital cellulitis	MALDI-TOF Mass Spectrometry	NR	NR	AMC < 0.016, AZY 2, CLI > 256, DAPTO 4, LZD 0.19, MEM 0.012, MTZ > 256, TZP < 0.016
Faiz (2025) [29]	Intraoperative sample from abscess	MALDI-TOF Mass Spectrometry (Bruker)	Kirby Bauer disk diffusion test	EUCAST 2023 breakpoint for *Corynebacterium*	CLI-, LIN-, RIF-, TET-susceptible CIP-, MOX-, PEN-, SXT-, VA-resistant
Lovering (2025) [30]	Blood culture	MALDI-TOF Mass Spectrometry	NR	NR	CN, CRO, LZD, MEM, PEN, VA susceptible
Saijo (2025) [31]	Intraoperative sample from debridement	MALDI-TOF Mass Spectrometry	NR	NR	CRO 0.5, FEP 2, CTX 0.5, ERY < 0.12, PEN 0.25, RIF < 1, SXT < 0.5, VA 0.5
Bozso (2024) [32]	Blood culture	NR	NR	NR	CLI ≤ 0.016, PEN ≤ 0.03
Chang (2024) [33]	Blood culture, intraoperative samples from abscess	NR	NR	NR	NR
Chin (2024) [34]	Intraoperative sample from abscess	NR	NR	NR	NR
Grusiecki (2024) [35]	Purulent material from abscess	NR	NR	NR	PEN-susceptible CIP-, CLI-, MXF-resistant
Gu (2024) [36]	Skin swab	MALDI-TOF Mass Spectrometry (Bruker)	Kirby Bauer disk diffusion test (Etest)	CLSI	PEN-susceptible
Lampejo (2024) [37]	Blood culture	MALDI-TOF Mass Spectrometry	Kirby Bauer disk diffusion test	EUCAST	CLI-, PEN-, VA-susceptible. MTZ-resistant
Alrwashdeh (2023) [38]	Blood culture and BAL	MALDI-TOF Mass Spectrometry		NR	PEN-susceptible
Herai (2023) [39]	Pleural fluid	MALDI-TOF Mass Spectrometry (Bruker)	NR	NR	NR
Sahhar (2023) [40]	Intraoperative sample from abscess	MALDI-TOF Mass Spectrometry (Bruker)	Kirby Bauer diffusion test (Disks and Etest)	NR	CLI-, CRO-, DOX-, LEV-, LZD-, PEN, RIF-, VA-susceptible
Hicks (2022) [41]	Intraoperative sample from abscess	MALDI-TOF Mass Spectrometry	Kirby Bauer diffusion test (Etest)	NR	PEN 0.064
Thomas (2022) [42]	Intraoperative sample from debridement	NR	NR	NR	NR
	Purulent material from wound	NR	NR	NR	NR
	Purulent material from wound	NR	NR	NR	NR
	Intraoperative sample from debridement	NR	NR	NR	NR
	Intraoperative sample from debridement	NR	NR	NR	NR
	Purulent material from ulcer	NR	NR	NR	NR
Adams (2020) [43]	Intraoperative sample from abscess	MALDI-TOF Mass Spectrometry	NR	NR	AMC-, AMP-, CTX-, ERY-, IPM-, LEV, MIN-, VA-susceptible CLI-resistant
Saeb (2021) [44]	Deep wound swab	16s RNA Sanger sequencing	NR	NR	NR
Lobo (2020) [45]	Purulent material from abscess	Biochemical identification (API Coryne Panel)	NR	NR	Quinolone susceptible
Verona (2020) [46]	Blood culture	Identification based on biochemical tests and MALDI-TOF Mass Spectrometry (Vitek MS)	Kirby–Bauer diffusion test (Etest)	CLSI breakpoint for infrequently isolated or fastidious bacteria (M45 CLSI 2015)	CN 2, PEN 0.023, VA 0.5
Takamura (2019) [3]	Blood culture	Biochemical identification (Walkerway and API Coryne Panel)	NR	CLSI breakpoint for Staphylococci	AMP 0.25, CFZ ≤ 8, CLI ≤ 0.5, CN 2, ERY ≤ 0.25, IPM ≤ 1, LEV ≤ 0.5, PEN 0.12, SAM ≤ 8, VA ≤ 0.5
Poplin (2018) [47]	Blood culture	MALDI-TOF Mass Spectrometry	NR	NR	NR
Seki (2019) [48]	Blood culture and biopsy of the tissue	Biochemical identification (BD Phoenix) and MALDI-TOF Mass Spectrometry	NR	NR	CLI ≤ 0.5, CN ≤ 4, CRO ≤ 0.5, ERY ≤ 0.5, FEP ≤ 0.5, IPM ≤ 0.25, LEV ≤ 2, PEN ≤ 1
Cortés-Penfield (2017) [49]	Blood culture and purulent material from abscess	Biochemical identification (RapID CB Plus Kit) and 16s rRNA sequencing	NR	NR	NR
Smith (2016) [50]	Blood culture and wound swab	Biochemical testing ANC card (VITEK2)	Not performed	Not performed	Not performed
Miyamoto (2015) [4]	Skin secretion	MALDI-TOF Mass Spectrometry (Bruker)	Broth microdilution	CLSI breakpoint (2010) for Corynebacterium	CLA > 32, CLI > 32, CN 2, CRO ≤ 0.25, IPM ≤ 0.25, LEV 0.5, MEM ≤ 0.25, MIN ≤ 0.25, PEN ≤ 0.06, VA 0.5
	Skin secretion				CLA ≤ 0.25, CLI ≤ 0.25, CN 2, CRO ≤ 0.25, IPM ≤ 0.25, LEV 0.5, MEM ≤ 0.25, MIN ≤ 0.25, PEN ≤ 0.06, VA 0.5
	Pus				CLA ≤ 0.25, CLI ≤ 0.25, CN > 32, CRO ≤ 0.25, IPM ≤ 0.25, LEV 0.5, MEM ≤ 0.25, MIN ≤ 0.25, PEN ≤ 0.06, VA 0.5
	Skin secretion				CLA ≤ 0.25, CLI ≤ 0.25, CN > 32, CRO ≤ 0.25, IPM ≤ 0.25, LEV 8, MEM ≤ 0.25, MIN ≤ 0.25, PEN ≤ 0.06, VA 0.5
	Pus				CLA ≤ 0.25, CLI ≤ 0.25, CN 2, CRO ≤ 0.25, IPM ≤ 0.25, LEV 8, MEM ≤ 0.25, MIN ≤ 0.25, PEN ≤ 0.06, VA 0.5
	Pus				CLA ≤ 0.25, CLI ≤ 0.25, CN 2, CRO ≤ 0.25, IPM ≤ 0.25, LEV 0.5, MEM ≤ 0.25, MIN ≤ 0.25, PEN ≤ 0.06, VA 0.5
	Skin secretion				CLA ≤ 0.25, CLI ≤ 0.25, CN 2, CRO ≤ 0.25, IPM ≤ 0.25, LEV 8, MEM ≤ 0.25, MIN ≤ 0.25, PEN ≤ 0.06, VA 0.5
Stone (2015) [51]	Intraoperative sample from debridement	NR	NR	NR	NR
Brown (2013) [52]	Blood culture and intraoperative tissue	Biochemical identification (RapID CB Plus Kit)	NR	NR	NR
Ji (2013) [53]	Blood culture	Biochemical testing (API CORYNE)	NR	NR	NR
Ramey (2013) [14]	Blood culture Orbital abscess culture	NR	NR	NR	CRO 0.064, ERY 0.032, PEN 0.032
Alatoom (2012) [54]	Postmortem blood culture	Biochemical testing (API CORYNE) and 16s RNA gene sequencing	NR	NR	NR
Hedman (2012) [15]	Wound swab from the foot Blood culture	NR	NR	NR	NR
	Blood culture				
	Blood culture				
Lee (2012) [55]	Blood culture	Biochemical testing (VITEK2) and 16s RNA gene sequencing	Kirby–Bauer disk diffusion test	CLSI breakpoint for *Streptococcus* spp.	CIP-, CLO-, CN-, VA-susceptible. CLI-, ERY-, PEN-, SXT-, TET-resistant
Sayyahfar (2012) [56]	Intraoperative purulent material from thyroid	NR	NR	NR	CIP-, CLI-, CN-, ERY-, FEP-, IPM-, VA-susceptible
Ribakovs (2011) [57]	Blood culture	NR	NR	NR	PEN-susceptible
Saxena (2011) [58]	Amniotic fluid	Identification based on biochemical tests	NR	NR	AMP-, CFL-, CIP-, CLI-, CN-, ERY-susceptible SXT-resistant
Wong (2011) [59]	Blood culture	Biochemical testing (API CORYNE) and 16s RNA gene sequencing	Kirby–Bauer disk diffusion test	NR	CIP-, CN-, CRO-, PEN-, RIF-, VA-susceptible
Lundblom (2010) [60]	Blood culture	16s RNA gene sequencing	NR	NR	NR
Fernández-Suárez (2009) [16]	Blood culture	Biochemical testing (API CORYNE)	Kirby–Bauer disk diffusion test	NR	CIP-, CLI-, CTX-, ERY-, OXA-, PEN-, TET-, VA-susceptible COL-resistant
Aracil (2008) [61]	Purulent discharge from the finger	Biochemical testing (API CORYNE)	Kirby–Bauer disk diffusion test	CLSI	AMP-, CFZ-, ERY-, LEV-, LIN-, VA-susceptible
Lee (2008) [17]	Wound swab from the left toe	Biochemical testing (API CORYNE)	Kirby–Bauer diffusion test (Etest)	CLSI	CLI 0.06 PEN 0.008
Malini (2008) [62]	Case 1 Pus sample from the ulcer	Identification based on the hemolytic pattern, catalase reaction and biochemical tests	NR	NR	CIP-, CLI-, CN-, ERY-, PEN-susceptible SXT-resistant
	Case 2 Pus sample from the ulcer				CIP-, CLI-, CN-, ERY-, PEN-susceptible SXT-resistant
	Case 3 Pus sample from the wound				CIP-, CLI-, CN-, ERY-, PEN-susceptible SXT-resistant
Therriault (2008) [1]	Blood culture, BAL and pus from abscess	16s RNA gene sequencing	Kirby–Bauer diffusion test (Etest)	CLSI breakpoint for *Staphylococcus aureus*	ERY < 0.016 PEN 0.023 VA 0.75
Volante (2008) [18]	Case 1 Intraoperative sample from abscess	NR	NR	NR	AMP, CRO, ERY, PEN-susceptible SXT resistant
	Case 2 Throat swab	NR	NR	NR	AZM, CIP, CLI, CRO, PEN susceptible SXT, VA resistant
Schroeder (2007) [63]	Blood culture and intraoperative tissue	NR	NR	NR	NR
van Loo (2007) [64]	Sample drained from abscess	Biochemical testing (API CORYNE)	Kirby–Bauer disk diffusion test	NR	CIP, SXT resistant
Ciraj (2006) [65]	Urine sample	Biochemical reactions	Kirby–Bauer disk diffusion test	NCCLS standards	AMK, AMC, AMP, CIP, CRO, ERY, PEN, VA susceptible
Farmer (2007) [66]	Sample from paracentesis	NR	NR	NR	ERY-susceptible
Tan (2006) [67]	Case 1 Blood culture Sample from the ulcer	NR	NR	NR	Four isolates were PEN-susceptible. Susceptibility testing not performed.
	Case 2 Blood culture Sample from abscess	NR	NR	NR	
	Case 3 Blood culture Sample from abscess	NR	NR	NR	
	Case 4 Blood culture Intraoperative tissue sample	NR	NR	NR	
	Case 5 Blood culture Sample from the ulcer	NR	NR	NR	
Vargas (2006) [68]	Intraoperative sample from abscess	Identification based on the hemolytic pattern, catalase reaction and biochemical tests	Kirby–Bauer disk diffusion test	NR	CLI-, CN-, CRO-, DOX-, PEN-, VA-susceptible CIP-, SXT-resistant
Goyal (2005) [69]	Synovial fluid	Identification based on biochemical tests	NR	NR	AMC-, CIP-, CRO-, ERY-, PEN-, VA-susceptible
Katkar (2005) [70]	Sputum	Identification based on the hemolytic pattern, catalase reaction and biochemical tests	NR	NR	NR
Parija (2005) [71]	Pus from thoracentesis	Identification based on the hemolytic pattern, catalase reaction and biochemical tests	Kirby–Bauer disk diffusion test	NR	AMP-, CIP-, CN-, CRO-, ERY-susceptible
Varma (2005) [72]	Purulent intraoperative sample	NR	NR	NR	NR

Abbreviations: NR: not reported; AMC: amoxicillin–clavulanate; AMK: amikacin; AMP: ampicillin; AZM: azithromycin; CLA: clarithromycin; CFL: cephalexin; CFZ: cefazolin; CIP: ciprofloxacin; CLI: clindamycin; CN: gentamycin; COL: colistin; CRO: ceftriaxone; CTX: cefotaxime; DAPTO: daptomycin; DOX: doxycycline; ERY: erythromycin; FEP: cefepime; IPM: imipenem; LEV: levofloxacin; LZD: linezolid; MEM: meropenem; MIN: minocycline; MTZ: metronidazole; MXF: moxifloxacin; OXA: oxacillin; PEN: penicillin; RIF: rifampin; SAM: ampicillin–sulbactam; SXT: trimethoprim–sulfamethoxazole; TET: tetracycline; TZP: piperacillin–tazobactam; VA: vancomycin.

### 3.4. Epidemiology of A. haemolyticum Infections

The epidemiology of *A. haemolyticum* infections reflects its dual role as both a community-associated pathogen in healthy adolescents and young adults, as well as an opportunistic pathogen in older or immunocompromised individuals. As shown in Table 1, cases were reported across a wide age range, with a predominance in adolescents and young adults for pharyngitis-related conditions, including peritonsillar abscesses and Lemierre’s syndrome. In contrast, invasive infections—such as bacteremia, endocarditis, necrotizing fasciitis, osteomyelitis, and intracerebral abscess—were more frequently described in older patients and in those with diabetes mellitus, cardiovascular disease, malignancies, or other chronic conditions. Polymicrobial infections were common, particularly in skin, soft tissue, and deep-seated infections, often involving anaerobes and other Gram-positive cocci. Data from reported cases show a predominance in male patients (Table 2), frequently in the second and third decades of life, particularly in pharyngitis-related conditions such as peritonsillar abscesses and Lemierre’s syndrome. Nonetheless, severe invasive disease—including bacteremia, endocarditis, necrotizing fasciitis, intracerebral abscesses, and osteomyelitis—has been increasingly described among older adults and patients with diabetes mellitus, cardiovascular disease, malignancies, or other chronic conditions. Co-infections are common, with *S. agalactiae*, *S. dysgalactiae*, *S. aureus* and anaerobic bacteria often isolated alongside *A. haemolyticum*, suggesting synergistic interactions in polymicrobial settings. The geographic distribution of cases indicates global occurrence, with reports spanning Europe, Asia, and North America, though the true burden is likely underestimated due to frequent misidentification as diphtheroids or streptococci in routine laboratories. However, the true burden of *A. haemolyticum* infections is likely underestimated due to frequent misidentification as diphtheroids or streptococci in routine clinical microbiology laboratories. Overall, these findings underscore the clinical heterogeneity of *A. haemolyticum* infections and the need for increased awareness of this pathogen in both community and hospital settings.

While pharyngitis remains the classic presentation, the wide spectrum of invasive infections highlights the organism’s capacity to affect diverse populations, with outcomes ranging from complete recovery after prolonged antimicrobial therapy to fatal complications in select cases.

### 3.5. Diagnosis of A. haemolyticum

Accurate diagnosis of *A. haemolyticum* remains challenging due to its slow growth, morphological similarity to coryneform bacteria, and frequent misidentification as diphtheroids or streptococci in routine laboratories. Early suspicion is crucial, particularly in patients with pharyngitis and scarlatiniform rash or invasive infections not explained by more common pathogens. Advances in diagnostic methods have improved detection, but awareness among clinicians and microbiologists is still limited.

#### 3.5.1. Identification of *A. haemolyticum*

Data regarding microbiological identification methods and antimicrobial susceptibility testing (AST) are summarized in Table 2. Traditional identification relies on colony morphology, β-hemolysis on human or horse blood agar, catalase negativity, and a positive reverse CAMP test. However, hemolysis may take up to 72 h to become apparent, leading to under-recognition in standard cultures. Recent case series demonstrate the increasing use of MALDI-TOF mass spectrometry (Maldi Biotyper (Bruker, Billerica, MA, USA), Vitek MS (bioMérieux, Marcy, l’Étoile, France), and other platforms) as a rapid and reliable method for species-level identification. In some cases, confirmatory testing with biochemical panels (API Coryne (bioMérieux, Marcy, l’Étoile, France), RapID CB Plus (Thermo Fisher Scientific, Waltham, MA, USA), BD Phoenix (Becton Dickinson and Company, Franklin Lakes, NJ, USA)) or molecular methods such as 16S rRNA sequencing has been necessary, particularly when cultures yielded mixed flora or atypical results. Despite these advances, the absence of standardized diagnostic algorithms contributes to variable recognition rates, and misclassification in polymicrobial infections remains a concern.

#### 3.5.2. Antimicrobial Susceptibility of *A. haemolyticum*

Antimicrobial susceptibility testing (AST) of *A. haemolyticum* is complicated by the lack of species-specific breakpoints, with laboratories commonly applying EUCAST or CLSI criteria for *Corynebacterium* or other fastidious Gram-positive organisms. AST was most commonly performed using the Kirby–Bauer disk diffusion method. Data from published cases show that most isolates remain susceptible to penicillin, ampicillin-sulbactam, and vancomycin, which form the mainstay of therapy. However, resistance has been variably reported to macrolides (erythromycin, clarithromycin), clindamycin, fluoroquinolones, trimethoprim–sulfamethoxazole, and, occasionally, rifampicin. Multidrug-resistant phenotypes, although rare, have been documented, including isolates resistant to penicillin and vancomycin. Co-infections with *Fusobacterium necrophorum*, *Streptococcus agalactiae*, or *Staphylococcus aureus* may further complicate susceptibility interpretation and therapeutic decision-making. These findings emphasize the importance of performing AST on all clinical isolates to guide therapy and highlight the emerging variability in resistance patterns among strains.

In Table 4, we suggest a laboratory approach for identification and AST of *A. haemolyticum* isolate.

## 4. Discussion, Treatment, and Outcomes of *A. haemolyticum* Infections

This narrative review provides an updated overview of the clinical spectrum, epidemiology, and microbiological characteristics of *A. haemolyticum* infections in humans, highlighting the persistent under-recognition of this pathogen in routine clinical practice. Although traditionally associated with pharyngitis in adolescents and young adults, the analysis of published cases demonstrates that *A. haemolyticum* is capable of causing a wide range of invasive and potentially life-threatening infections.

As shown in Table 1, skin and soft-tissue infections and bloodstream infections represented the most frequently reported clinical manifestations, followed by Lemierre’s syndrome and central nervous system involvement. These findings confirm that *A. haemolyticum* should no longer be regarded as a pathogen limited to benign upper respiratory tract infections. Instead, it should be considered a clinically relevant organism with the ability to invade deep tissues and cause systemic disease, particularly in the presence of predisposing factors such as diabetes mellitus, malignancy, or other chronic conditions. A notable finding of this review is the high proportion of polymicrobial infections. *A. haemolyticum* was frequently isolated alongside β-hemolytic streptococci, anaerobic bacteria, and Staphylococcus aureus, suggesting that it may act synergistically with other pathogens, especially in skin, soft tissue, and deep-seated infections. This observation aligns with previous hypotheses proposing that toxin production—most notably phospholipase D—may facilitate tissue invasion and enhance the pathogenicity of co-infecting organisms. The frequent involvement of anaerobes further underscores the importance of considering broad-spectrum antimicrobial coverage in severe infections until microbiological results become available.

From a diagnostic perspective, this review highlights substantial heterogeneity in laboratory identification methods over time. Earlier studies relied predominantly on phenotypic characteristics, which are often insufficient to reliably distinguish *A. haemolyticum* from other Gram-positive bacilli or β-hemolytic streptococci. The increasing adoption of MALDI-TOF mass spectrometry, as reported in more recent cases (Table 2), represents a major advance in diagnostic accuracy and is likely to contribute to improved detection rates. Nevertheless, misidentification remains a concern, particularly in laboratories without access to advanced diagnostic tools. Antimicrobial susceptibility data revealed considerable variability. While most isolates remained susceptible to β-lactam antibiotics, resistance to macrolides, clindamycin, fluoroquinolones, and trimethoprim–sulfamethoxazole was documented, and sporadic resistance to vancomycin was also reported. These findings are clinically relevant, as macrolides are frequently used for empirical treatment of pharyngitis and skin infections. The lack of standardized AST breakpoints specific for *Arcanobacterium* spp. further complicates therapeutic decision-making and underscores the need for susceptibility testing whenever feasible. The global distribution of reported cases suggests that *A. haemolyticum* infections occur worldwide. However, the true incidence is likely underestimated due to diagnostic challenges and limited awareness among clinicians and microbiologists. Increased recognition of this pathogen, combined with improved laboratory identification, may reveal a higher burden of disease than currently appreciated. This review has several limitations. The analysis is based primarily on case reports and small case series, which are subject to publication bias and incomplete reporting. In addition, heterogeneity in diagnostic methods and antimicrobial susceptibility testing limits the ability to draw firm conclusions regarding resistance patterns. Despite these limitations, the collected data provide valuable insights into the evolving clinical relevance of *A. haemolyticum.*

## 5. Conclusions

*Arcanobacterium haemolyticum* is an underdiagnosed but clinically significant pathogen capable of causing a broad spectrum of infections, ranging from mild pharyngitis to severe invasive disease. The findings of this review emphasize its frequent involvement in polymicrobial infections, its association with both immunocompetent and immunocompromised hosts, and the growing relevance of antimicrobial resistance. Improved awareness, accurate laboratory identification—particularly through MALDI-TOF mass spectrometry—and routine antimicrobial susceptibility testing are essential to optimizing patient management. Given the absence of standardized AST guidelines for this organism, further studies are needed to better define susceptibility patterns and inform evidence-based therapeutic strategies. Overall, *A. haemolyticum* should be systematically considered in the differential diagnosis of invasive and polymicrobial infections to reduce the risk of delayed or inappropriate treatment.

## Figures and Tables

**Table 1:** Summary of *Arcanobacterium haemolyticum* infections reported in the literature.

**Gender**	50 M; 23 F
**Age**	Range: 2–91 (M: 7–81; F: 2–91) Average: 44.5 (M: 41.6; F: 50.9) IQR: 21–64 (M: 20.3–61.5; F: 31–66.5)
Type of infection *	Bloodstream infection, *n* = 19 Skin and soft tissue infection, *n* = 19 Brain abscess, *n* = 6 Lemierre’s syndrome, *n* = 6 Bone infection, *n* = 4 Intra-abdominal infection, *n* = 4 Respiratory tract infection, *n* = 4 Pyothorax, *n* = 3 Endocarditis, *n* = 2 Urinary tract infection, *n* = 1 Other type of infection, *n* = 5
**Monomicrobial infection**	40 samples **
**Polymicrobial infection**	32 samples (9 patients with more than one samples, at least one was polymicrobial. Some samples had more than one co-isolated bacteria) β-hemolytic streptococci, *n* = 16 Anaerobic bacteria, *n* = 12 *S. aureus*, *n* = 10

Abbreviations: F: Female; M: Male. * Classification based on the most severe infection to treat. ** One sample with PCR positive for other microorganisms but with cultural analysis positive only for *A. haemolyticum* was considered as monomicrobial infection (in one case information about co-infection with other organisms was not reported).

**Table 4:** Laboratory approach for identification and AST of *A. haemolyticum*.

**Incubation** **Conditions**	24–72 h at 35 ± 1 °C in 5% CO_2_ blood sheep agar media. Hemolysis became more evident after 48–72 h of incubation
**Identification**	MALDI-TOF MS has very good performance in identification of *A. haemolyticum*.
**AST** **(Agar diffusion method)**	Media: Mueller–Hinton agar + 5% defibrinated horse blood and 20 mg/L β-NAD (MH-F). Inoculum: McFarland 0.5. Incubation: 5% CO_2_, 35 ± 1 °C, 18 ± 2 h.
**Interpretation** **criteria**	EUCAST Guidance document “When there are no breakpoints in breakpoint tables?” published in September 2024 for Gram-positive organisms [73].
**Suggested** **Antimicrobial** **agents**	Benzylpenicillin Ampicillin–sulbactam and/or amoxicillin–clavulanic acid Vancomycin Clindamycin Ciprofloxacin and/or levofloxacin Trimethoprim–sulfamethoxazole Rifampicin

## Data Availability

No new data were created or analyzed in this study. Data sharing is not applicable to this article.

## References

[1] Therriault B.L., Daniels L.M., Carter Y.L., Raasch R.H. (2008). Severe sepsis caused by *Arcanobacterium haemolyticum*: A case report and review of the literature. Ann. Pharmacother..

[2] Kang H., Park G., Kim H., Chang K. (2016). Haemolytic differential identification of *Arcanobacterium haemolyticum* isolated from a patient with diabetic foot ulcers. JMM Case Rep..

[3] Takamura N., Tada K., Ishioka H., Gomi H. (2019). Clinically Infrequent *Arcanobacterium haemolyticum* Bacteremia Complicated by Foot Decubitus Ulcer: An Educational Reminder for Primary Care Physicians. Intern. Med..

[4] Miyamoto H., Suzuki T., Murakami S., Fukuoka M., Tanaka Y., Kondo T., Nishimiya T., Suemori K., Tauchi H., Osawa H. (2015). Bacteriological characteristics of *Arcanobacterium haemolyticum* isolated from seven patients with skin and soft-tissue infections. J. Med. Microbiol..

[5] Sayad E., Zeid C.A., Hajjar R.E., Cabrera N.L., Radi Abou Jaoudeh R.A., Malek A.E. (2021). The burden of *Arcanobacterium haemolyticum* pharyngitis: A systematic review and management algorithm. Int. J. Pediatr. Otorhinolaryngol..

[6] Miller R.A., Brancato F., Holmes K.K. (1986). *Corynebacterium hemolyticum* as a cause of pharyngitis and scarlatiniform rash in young adults. Ann. Intern. Med..

[7] Carlson P., Seppänen M., Tarvainen K., Nousiainen T., Aaltonen T., Malinen H. (2001). Pharyngitis and exanthema caused by *Arcanobacterium haemolyticum*. Acta Derm. Venereol..

[8] Collins M.D., Jones D. (1982). Reclassification of *Corynebacterium pyogenes* (Glage) in the genus *Actinomyces*, as *Actinomyces pyogenes* comb.nov. J. Gen. Microbiol..

[9] MacLean P.D., Liebow A.A., Rosenberg A.A. (1946). A hemolytic *Corynebacterium* resembling *Corynebacterium ovis* and *Corynebacterium pyogenes* in man. J. Infect. Dis..

[10] Ramos C.P., Foster G., Collins M.D. (1997). Phylogenetic analysis of the genus *Actinomyces* based on 16S rRNA gene sequences: Description of *Arcanobacterium phocae* sp. nov., *Arcanobacterium bernardiae* comb. nov., and *Arcanobacterium pyogenes* comb. nov. Int. J. Syst. Bacteriol..

[11] Lehnen A., Busse H.J., Frolich K., Krasinska M., Kampfer P., Speck S. (2006). *Arcanobacterium bialowiezense* sp. nov. and *Arcanobacterium bonasi* sp. nov., isolated from the prepuce of European bison bulls (*Bison bonasus*) suffering from balanoposthitis, and emended description of the genus *Arcanobacterium* Collins et al. 1983. Int. J. Syst. Evol. Microbiol..

[12] Carlson P., Lounatmaa K., Kontiainen S. (1994). Biotypes of *Arcanobacterium haemolyticum*. J. Clin. Microbiol..

[13] Balıkcı A., Topkaya A.E., Belaş Z. (2011). [A frequently overlooked bacteria in clinical microbiology laboratories: *Arcanobacterium haemolyticum*]. Mikrobiyol. Bul..

[14] Ramey N.A., Burkat C.N. (2013). *Arcanobacterium hemolyticum* orbital cellulitis: A rare but aggressive disease. Ophthalmic Plast. Reconstr. Surg..

[15] Hedman K., Brauner A. (2012). Septicaemia caused by *Arcanobacterium haemolyticum* smooth type in an immunocompetent patient. J. Med. Microbiol..

[16] Fernández-Suárez A., Aguilar Benítez J.M., López Vidal A.M., Díaz Iglesias J.M. (2009). Lemierre’s syndrome and septicaemia caused solely by *Arcanobacterium haemolyticum* in a young immunocompetent patient. J. Med. Microbiol..

[17] Lee S., Roh K.H., Kim C.K., Yong D., Choi J.Y., Lee J.W., Lee K., Chong Y. (2008). A case of necrotizing fasciitis due to *Streptococcus agalactiae*, *Arcanobacterium haemolyticum*, and *Finegoldia magna* in a dog-bitten patient with diabetes. Korean J. Lab. Med..

[18] Volante M., Corina L., Contucci A.M., Calò L., Artuso A. (2008). *Arcanobacterium haemolyticum*: Two case reports. Acta Otorhinolaryngol. Ital..

[19] Soucek A., Souckova A. (1974). Toxicity of bacterial sphingomyelinases D. J. Hyg. Epidemiol. Microbiol. Immunol..

[20] Votava M., Skalka B., Woznicova V., Ruzicka F., Zahradnicek O., Ondrovcik P., Klapacova L. (2001). Detection of *Arcanobacterium haemolyticum* phospholipase D neutralizing antibodies in patients with acute tonsillitis. Epidemiol. Mikrobiol. Imunol..

[21] Skalka B., Literak I., Chalupa P., Votava M. (1998). Phospholipase D-neutralization in serodiagnosis of *Arcanobacterium haemolyticum* and *Corynebacterium pseudotuberculosis* infections. Zentralbl. Bakteriol..

[22] Sengupta P., Baird B., Holowka D. (2007). Lipid rafts, fluid/fluid phase separation, and their relevance to plasma membrane structure and function. Semin. Cell Dev. Biol..

[23] Zhang Y., Li X., Becker K.A., Gulbins E. (2009). Ceramide-enriched membrane domains—Structure and function. Biochim. Biophys. Acta.

[24] Cuevas W.A., Songer J.G. (1993). *Arcanobacterium haemolyticum* phospholipase D is genetically and functionally similar to *Corynebacterium pseudotuberculosis* phospholipase D. Infect. Immun..

[25] McNamara P.J., Bradley G.A., Songer J.G. (1994). Targeted mutagenesis of the phospholipase D gene results in decreased virulence of *Corynebacterium pseudotuberculosis*. Molec Microbiol..

[26] Tambourgi D.V., De Sousa Da Silva M., Billington S.J., Goncalves De Andrade R.M., Magnoli F.C., Songer J.G., Van Den Berg C.W. (2002). Mechanism of induction of complement susceptibility of erythrocytes by spider and bacterial sphingomyelinases. Immunology.

[27] Yozwiak M.L., Songer J.G. (1993). Effect of *Corynebacterium pseudotuberculosis* phospholipase D on viability and chemotactic responses of ovine neutrophils. Am. J. Vet. Res..

[28] Chinello P., Capone A., Al Moghazi S., Cirillo P., Fontana C., Cicalini S. (2025). Periorbital and Central Nervous System Infection Due to *Arcanobacterium haemolyticum*: Case Report and Review of the Literature. Microorganisms.

[29] Faiz I., Saddari A., Ezrari S., Kaddouri S., Benaissa E., BenLahlou Y., Elouennass M., Maleb A. (2025). Acute appendicitis caused by *Arcanobacterium haemolyticum*. Clin. Microbiol. Newsl..

[30] Lovering E., Bahrain M. (2025). *Arcanobacterium haemolyticum* Chronic Osteomyelitis and Bacteremia Complicated by Intraosseous Abscess: A Case Report. J. Community Hosp. Intern. Med. Perspect..

[31] Saijo M., Constantine S., Wakai T., Wada N., Narita M. (2025). A Case of Necrotizing Fasciitis Caused by Mixed Infection of *Arcanobacterium haemolyticum* and *Streptococcus agalactiae*. Cureus.

[32] Bozso C.R., Leibowitz J., Barton M., Ostovar G.A., Rubin L.G. (2024). *Arcanobacterium haemolyticum* Bacteremia and Possible Meningitis in a Previously Healthy 14-Year-Old Male Patient: A Case Report. Infect. Dis. Clin. Pract..

[33] Chang N., Lennard K., Rao A., Elliott M., Dharan N., Wong J. (2024). Polymicrobial arcanobacterium haemolyticum intracerebral abscess: A case report and review of the literature. IDCases.

[34] Chin S.J., Horton D. (2024). Double whammy: Delayed cerebral ischemia of a 19-year-old secondary to sinogenic complications from an uncommon bacterial sinusitis, *Arcanobacterium haemolyticum*. Radiol. Case Rep..

[35] Grusiecki D., Marcinkiewicz B., Zawadzka-Głos L. (2024). *Arcanobacterium haemolyticum*—Case report. New Med..

[36] Gu Y., Millan G., Sebaratnam D.F. (2024). *Arcanobacterium haemolyticum*—Infection in an Immunocompetent Child. Pediatr. Infect. Dis. J..

[37] Lampejo T., Alsheikh F., Crilly D., Brown M. (2024). Lemierre’s syndrome: Varying pathogens, clinical presentations and complications. Diagn. Microbiol. Infect. Dis..

[38] Alrwashdeh A.M., Saluja P., Hasan L., Kocurek E., Dare R.K. (2023). *Arcanobacterium haemolyticum* bacteremia presenting as severe sepsis: A case report and review of the literature. IDCases.

[39] Herai Y., Nakahara T., Kasai K., Omori S., Tokuhiro K. (2023). Pyothorax Caused by *Arcanobacterium haemolyticum* and *Staphylococcus aureus* Co-infection: A Case Report. Cureus.

[40] Sahhar H.S., Rubin E., Rishmawi S.E., Logan M. (2023). Invasive Sinusitis with *Arcanobacterium haemolyticum* and *Fusobacterium necrophorum* Complicated by Subdural Empyema in an Immunocompetent Adolescent Patient. Cureus.

[41] Hicks S.R., Banavathi K. (2022). Necrotising faciitis caused by *Arcanobacterium haemolyticum*. Clin. Infect. Pract..

[42] Thomas T., Gachinmath S., Kumari P. (2022). *Arcanobacterium haemolyticum*: A case series. Trop. Doct.

[43] Adams N., Snitchler C., Kong M., Ikeda D., Skinner A., Rodriguezbarrantes J., Leverette R., Bell R. (2020). When upper respiratory tract infections go rogue: A case report of *Arcanobacterium haemolyticum* Cerebral Abscess. IDCases.

[44] Saeb A.T.M., Al-Rubeaan K.A., Mani B., Tayeb H.T. (2021). Osteomyelitis infection caused by *Arcanobacterium haemolyticum* in a diabetic patient: A first case report. IDCases.

[45] Lobo J., Gallo C., Mejuto P. (2020). Liver abscess caused by *Arcanobacterium haemolyticum*. Rev. Esp. Enferm. Dig..

[46] Verona J., Dirialdi N.C., Mutti M.V., Scioli M.C., Thougnon Islas F.A., Curi Antún G.E., Sgariglia L.J., Homar M.G., Legato J.A., Costa A. (2020). Bacteremia and sepsis by *Arcanobacterium haemolyticum* in a young immunocompetent patient. Rev. Argent. Microbiol..

[47] Poplin V., McKinsey D.S. (2018). Arcanobacterium Brain Abscesses, Subdural Empyema, and Bacteremia Complicating Epstein-Barr Virus Mononucleosis. Kans. J. Med..

[48] Seki Y., Morii D., Hata K., Unno T., Yokozawa T., Li S., Oda T. (2019). A Case Report of an Intrathoracic Mass Lesion Caused by *Arcanobacterium haemolyticum* that Required Exclusion of a Malignant Tumor Diagnosis. JMA J..

[49] Cortés-Penfield N., Kohli A., Weatherhead J., El Sahly H. (2017). *Arcanobacterium haemolyticum* CNS abscess and bacteremia following head trauma: A case report and literature review. Infect. Dis. Clin. Pract..

[50] Smith H.M., Arul I. (2016). Wound Infection and Sepsis Caused by *Arcanobacterium haemolyticum*. Clin. Microbiol. Newsl..

[51] Stone L.A., Harshbarger R.J. (2015). Orbital necrotizing fasciitis and osteomyelitis caused by *arcanobacterium haemolyticum*: A case report. Ophthalmic Plast. Reconstr. Surg..

[52] Brown J., Fleming C.S., Troia-Cancio P.V. (2013). *Arcanobacterium haemolyticum* osteomyelitis and sepsis: A diagnostic conundrum. Surg. Infect..

[53] Ji Y.Q., Wang J., Kong L.Q., Juggessur-Mungur K.S., Wu T.H., Zhang Z.H. (2013). Lemierre syndrome caused by *Arcanobacterium haemolyticum*. Chin. Med. J..

[54] Alatoom A., Duval J.V., Cavuoti D. (2012). Fatal Endocarditis Caused by *Arcanobacterium haemolyticum*: Case Report and Review of the Literature. Clin. Microbiol. Newsl..

[55] Lee K.J., Kim E.J., Kang S.J., Jang M.O., Jang H.C., Jung S.I., Shin J.H., Park K.H. (2012). Lemierre Syndrome Caused by *Arcanobacterium haemolyticum* Alone in a Healthy Man. Chonnam Med. J..

[56] Sayyahfar S., Nasiri S.J. (2012). First report of a thyroid abscess in the pediatric age group caused by *Arcanobacterium haemolyticum*. J. Infect. Chemother..

[57] Ribakovs A., Uzoigwe C.E., Bhansali H. (2011). Sequential *Arcanobacterium haemolyticum* and *Escherichia coli* spinal infections after local excision of a rectal polyp. Am. Surg..

[58] Saxena S., Aggarwal C., Anuradha Mehta G. (2011). Chorioamnionitis due to *Arcanobacterium haemolyticum*. J. Glob. Infect. Dis..

[59] Wong V., Turmezei T., Cartmill M., Soo S. (2011). Infective endocarditis caused by *Arcanobacterium haemolyticum*: A case report. Ann. Clin. Microbiol. Antimicrob..

[60] Lundblom K., Jung K., Kalin M. (2010). Lemierre syndrome caused by co-infection by *Arcanobacterium haemolyticum* and *Fusobacterium necrophorum*. Infection.

[61] Aracil B., Buzón L., Varela M., Olivas J., Gómez L. (2008). Osteomyelitis Caused by *Arcanobacterium haemolyticum* in the Distal Phalanx of a Thumb. Clin. Microbiol. Newsl..

[62] Malini A., Deepa E.K., Manohar P.V., Borappa K., Prasad S.R. (2008). Soft tissue infections with *Arcanobacterium haemolyticum*: Report of three cases. Indian. J. Med. Microbiol..

[63] Schroeder J. (2007). An unusual presentation of Lemierre syndrome caused by *Arcanobacterium haemolyticum*. Infect. Med..

[64] van Loo I.H.M., van den Wildenberg W.J., van Huijstee P.J., Roukema J.A., Apperloo A.J., Peeters M.F. (2007). Pelvic abscess caused by *Arcanobacterium haemolyticum* mimicking a soft tissue tumour. J. Med. Microbiol..

[65] Ciraj A.M., Rajani K., Sreejith G., Shobha K.L., Rao P.S. (2006). Urinary tract infection due to *Arcanobacterium haemolyticum*. Indian. J. Med. Microbiol..

[66] Farmer A.D., Bruckner Holt C.E., Le Roux G., Butterworth J.R. (2007). Spontaneous bacterial peritonitis due to *Arcanobacterium haemolyticum*. J. Infect..

[67] Tan T.Y., Ng S.Y., Thomas H., Chan B.K. (2006). *Arcanobacterium haemolyticum* bacteraemia and soft-tissue infections: Case report and review of the literature. J. Infect..

[68] Vargas J., Hernandez M., Silvestri C., Jiménez O., Guevara N., Carballo M., Rojas N., Riera J., Alayo E., Fernández M. (2006). Brain abscess due to *Arcanobacterium haemolyticum* after dental extraction. Clin. Infect. Dis..

[69] Goyal R., Singh N.P., Mathur M. (2005). Septic arthritis due to *Arcanobacterium haemolyticum*. Indian J. Med. Microbiol..

[70] Katkar V.J., Shrikhande S.N., Deshpande A., Tankhiwale N.S. (2005). Respiratory tract infection due to *Arcanobacterium haemolyticum*—A case report. Indian. J. Pathol. Microbiol..

[71] Parija S.C., Kaliaperumal V., Kumar S.V., Sujatha S., Babu V., Balu V. (2005). *Arcanobacterium haemolyticum* associated with pyothorax: Case report. BMC Infect. Dis..

[72] Varma D., Chang B., Musaad S. (2005). A case series on chronic canaliculitis. Orbit.

[73] When There Are No Breakpoints in the Breakpoints Table. https://www.eucast.org/fileadmin/eucast/pdf/guidance_documents/When_there_are_no_breakpoints_2024-09-03.pdf.

